# Novel Antioxidant and Hypoglycemic Water-Soluble Polysaccharides from Jasmine Tea

**DOI:** 10.3390/foods10102375

**Published:** 2021-10-07

**Authors:** Yayuan Tang, Jinfeng Sheng, Xuemei He, Jian Sun, Zhen Wei, Guoming Liu, Changbao Li, Bo Lin, Li Li

**Affiliations:** 1 Agro-Food Science and Technology Research Institute, Guangxi Academy of Agricultural Sciences, 174 East Daxue Road, Nanning 530007, China; tangyayuan@gxaas.net (Y.T.); shengjinfeng@gxaas.net (J.S.); zhenwei@gxaas.net (Z.W.); guoming-liu@gxaas.net (G.L.); changbaoli@gxaas.net (C.L.); linbo@gxaas.net (B.L.); lili@gxaas.net (L.L.); 2 Guangxi Key Laboratory of Fruits and Vegetables Storage-Processing Technology, 174 East Daxue Road, Nanning 530007, China; 3 Guangxi Academy of Agricultural Sciences, 174 East Daxue Road, Nanning 530007, China

**Keywords:** *Jasminum sambac* (L.) Aiton tea, polysaccharides, antioxidant capacity, anti-hyperglycemic activity, structural analysis

## Abstract

There have been few studies dealing with chemical elucidation and pharmacological potentials of water-soluble polysaccharides from jasmine tea, limiting their use in functional foods. In this study, water-soluble polysaccharides (named as JSP) were extracted from *Jasminum sambac* (L.) Aiton tea and fractionated to afford two sub-fractions (JSP-1 and JSP-2). The main structural characteristics of novel JSP sub-fractions were determined by high performance gel permeation chromatography, ultra-performance liquid chromatography-tandem mass spectrometry, Fourier transform infrared, and nuclear magnetic resonance analysis. Physiologically, the abilities of JSP-1 and JSP-2 to reduce ferric ions, scavenge DPPH and hydroxyl radicals, as well as protect islet cells were confirmed in vitro. JSP-1 exhibited better antioxidant and hypoglycemic activities than JSP-2. The molecular weights of JSP-1 and JSP-2 were 18.4 kDa and 14.1 kDa, respectively. JSP-1 was made up of glucose, galactose, rhamnose, xylose, arabinose, and galacturonic acid with molar ratios 1.14:4.69:1.00:9.92:13.79:4.09, whereas JSP-2 with a triple helical structure was composed of galactose, rhamnose, xylose, arabinose, and galacturonic acid as 3.80:1.00:8.27:11.85:5.05 of molar ratios. JSP-1 contains →1)-α-Gal*ƒ*-(3→, →1)-α-Gal*ƒ*-(2→, →1)-α-Ara*ƒ*-(5→, →1)-α-Ara*ƒ*-(3→, →1)-α-Ara*ƒ*-(3,5→, →1)-β-Xyl*p*-(2→ and →1)-β-Xyl*p*-(3→ residues in the backbone. These results open up new pharmacological prospects for the water-soluble polysaccharides extracted from jasmine tea.

## 1. Introduction

Diabetes mellitus, as the third most life-threatening illnesses worldwide, is a syndrome of endocrine metabolic disorder characterized by sustained hyperglycemia. The high concentration of free radicals induced by chronic hyperglycemia can damage biological macromolecules in the islet cells and the pancreatic tissues, increasing the risk of diabetes. Thus, protecting the islet cells from oxidative stress appears to be a crucial factor to lower blood glucose and successfully prevent diabetes [1]. Nowadays, many edible and medicinal polysaccharides attract attention based on their special physico-chemical properties and physiological functions, including antioxidant and hypoglycemic activities [2]. The particular bioactivity of polysaccharides is linked to structural properties such as molecular weight, monosaccharide content, functional group and three-dimensional conformation, as well as glycosidic linkage [3].

The genus *Jasminum* belonging to the *Oleaceae* family is a kind of bulb flower with high potentials [4]. *Jasminum sambac* (L.) Aiton is mainly cultivated in tropical regions in Asia and warm temperate regions in Europe and Africa for their attraction and delightfully fragrant blossoms. *J. sambac* is a well-known medicinal plant that appears in over 30% of traditional Chinese Medicine and folk medicine formulas for anti-inflammatory, anti-hyperlipidemic, anti-diabetic, anti-depressant, and analgesic treatments as well as wound healing [5,6,7]. Jasmine tea is the major value-added processed product of *J. sambac*, accounting for approximately 80% of the whole *J. sambac* processed products in the food industry. Various active ingredients found in jasmine tea such as flavonoids, polysaccharides, and essential oils have antioxidant, anti-microbial, anti-neoplastic, anti-diabetic, and regulating immunological properties according to extensive studies. The crude extracts of jasmine tea showed free radicals scavenging activities [8,9]. As compared to diabetic control, blood glucose content in jasmine tea-isolated treated animals was much lower than in oral glucose resistance tests as well as alloxan-induced diabetes and streptozotocin-induced diabetes models [10]. Jasmine tea extracts can ameliorate insulin resistance in both high fat diet-induced rats and streptozotocin-induced rats as well. Moreover, tea polysaccharides have the advantages of high safety and low toxicity in traditional use, including protecting islet cells, increasing the number of islet cells, promoting insulin secretion or release, and improving glucose metabolism in treating diabetes. Crude polysaccharides from jasmine tea had regulation effects on immune responses in alloxan-induced diabetes rats. Owing to these benefits, bioactive compounds found in jasmine tea have been used widely in functional foods, as main ingredients in natural preservatives and detergents for fruits and vegetables, and as aromatizing agents in the food industry.

Despite this, to our knowledge, there have been no studies dealing with the chemical elucidation and the pharmacological potentials of water-soluble polysaccharides from jasmine tea, limiting their use in functional foods. Accordingly, the objective of this study was to explore antioxidant (total antioxidant capacity and •OH and DPPH• scavenging abilities) and hypoglycemic activities in vitro of polysaccharide sub-fractions from jasmine tea. Subsequently, some structural features of main JSP sub-fractions were preliminarily identified by high performance gel permeation chromatography (HPGPC), ultra-performance liquid chromatography-tandem mass spectrometry (UPLC-MS), ultraviolet (UV), Fourier transform infrared spectroscopy (FTIR), and nuclear magnetic resonance (NMR) analysis. In view of the above facts, this paper further provides a reference for the potential utilization of jasmine tea polysaccharides in food industries.

## 2. Materials and Methods

### 2.1. Chemicals and Reagents

RIN-m5F (ATCC CRL-11605) cells were purchased from the Tongpai Biotechnology Co. Ltd., Shanghai, China. Cytiva (Uppsala, Sweden) provided diethylaminoethyl 52 (DEAE-52) cellulose and Sephadex G-100. Sodium chloride, anthrone, D-glucose, penicillin-streptomycin solution (100×, including penicillin 10 ku/mL and streptomycin 10 mg/mL), phosphate buffer solution (1×, pH 7.2–7.4, 0.01 M), trypsin-EDTA solution, and Total Antioxidant Capacity (T-AOC) Assay Kit were purchased from Beijing Solarbio Science and Tech. Co. (Beijing, China). Gibco RPMI medium 1640 basic (1×) was arranged from ThermoFisher Biochemical Products (Beijing) Co. (Beijing, China). Fetal bovine serum (FBS, Australia Origin) and Cell Counting Kit-8 were arranged by Invigentech Inc. (Irvine, CA, USA). Sigma-Aldrich Co. (St. Louis, MO, USA) provided the 2-diphenyl-1-picrylhydrazyl (DPPH). Nanjing Jiancheng Bioengineering Institute (Nanjing, Jiangsu, China) provided the Hydroxyl Free Radical Assay Kit. Absolute ethanol, chloroform, n-butanol, concentrated sulfuric acid, and potassium bromide were purchased from the Tianjin Damao Chemical Reagent Co. (Tianjin, China). All compounds were analytical grade unless otherwise specified.

### 2.2. Preparation of Jasmine Tea

Raw *J. sambac* flowers were harvested in the month of May 2020 on the farm of Shunlai Tea Industry Co. Ltd. located in Hengxian County, Guangxi Province, China. These flowers were ground into fine powder in an FW 177 high rotational speed disintegrator (Taisite Instrument, Tianjin, China) after drying for 6 h (60 °C) in a WGLL-230BE electro-thermostatic blast oven (Taisite Instrument, Tianjin, China). The powder was sieved through an 80# mesh and stored in a cool, dry environment for further analysis.

### 2.3. Extraction, Isolation, and Purification of Crude Polysaccharides from Jasmine Tea

Crude polysaccharides in jasmine tea were extracted and isolated according to the procedure of Eakwaropas and Wisidsri (2019) with minor changes [11]. The dried *J. sambac* flower powder (300 g) was extracted in a ratio of 1:30 (*w/v*) for 3 h using hot water in a 90 °C HH-S6 water bath (Jinan OLABO Technology Co. Ltd., Jinan, Shandong province, China). The extraction was carried out twice. A RE-5205 rotatory evaporator (Shanghai Yarong Biochemistry Instrument Factory, Shanghai, China) was used to concentrate supernatants at 60 °C. The concentrate was freeze-dried after being precipitated with 4 times the volume of ethanol at 4 °C for 24 h (Labconco Corporation, Kansas City, MO, USA). The precipitate was diluted in distilled water and then deproteinated by the procedure of Sevag (chloroform: n-butanol = 4:1, *v/v*) [12], followed by 3 days of dialysis with the Minimate TFF capsule 3K Omega membrane (Pall Corporation, Port Washington, NY, USA) to remove oligosaccharides or monosaccharides and contaminants. The crude polysaccharides of *J. sambac* flower (JSP) were finally obtained.

A two-step purification technique was used to purify the crude JPS [13]. Initially, a DEAE-52 cellulose column (2.6 cm × 50 cm) was used to apply crude-JSP solution. The mobile phase was used at ultra-pure water and various levels of NaCl (0.1, 0.3, and 0.5 M) at a flow rate of 1 mL/min, respectively. Each 5 mL of eluent for one tube was collected by a DBS-100 automatic collector (Shanghai Huxi Analysis Instrument Factory Co., Shanghai, China), which was detected by the anthrone-sulfuric acid method to obtain the elution profile. The two major fractions were collected and dialyzed with Minimate TFF capsule 3K Omega membrane by PALL Minimate Tangential Flow Filtration Systems (Port Washington, NY, USA) for 3–4 d, respectively. These fractions were concentrated using the RE-5205 rotatory evaporator and then freeze-dried by a freeze-drier (Labconco Corporation, Kansas City, MO, USA) for 48 h. Then, the resultant main fractions were subjected to a Sephadex G-100 column (2.6 cm × 50 cm) and eluted at 0.5 mL/min flow rate. The eluents (5 mL per tube) were collected and analyzed as previously described. Total carbohydrates were quantified using the method of anthrone-sulfuric acid with glucose as the reference [14].

### 2.4. Functional Activities of JSP Sub-Fractions

#### 2.4.1. Antioxidant Activities

T-AOC, DPPH radical scavenging capacity, and hydroxyl radical (•OH) scavenging activities were used to determine the antioxidant properties of JSP sub-fractions. A positive control was employed, which was ascorbic acid. T-AOC was determined using the Total Antioxidant Capacity (T-AOC) Assay Kit. The T-AOC value was calculated using micromoles of Fe^2+^ equivalents (Fe) per mL of JSP sub-fractions and a calibration standard curve of FeSO_4_•7H_2_O solution. A colorimetric approach was used to measure the DPPH free radical scavenging ability [15]. In brief, different levels (0.02–1.50 mg/mL) of JSP sub-fractions (0.1 mL) were mixed with DPPH-ethanol solution (0.1 mL and 0.1 mM) and left for 30 min in the dark. An EpochTM microplate spectrophotometer (BioTek Instruments, Inc., Winooski, VT, USA) was used to record the absorbance at 517 nm. The percentage of DPPH discoloration (%) = [A_blank_ − (A_sample_ − A_control_)]/A_blank_ × 100, where A_sample_ represented the absorbance of the solution with DPPH and JSP sub-fractions; A_control_ was the absorbance of the solution with JSP sub-fractions and ethanol; and A_blank_ represented the absorbance of the solution with DPPH and ethanol. Additionally, •OH was analyzed using a Hydroxyl Free Radical Assay Kit.

#### 2.4.2. Hypoglycemic Effect

##### Cell Culture

Hyperglycemic rat pancreatic islet (RIN-m5F) cells were cultured in RPMI 1640 with fetal bovine serum (10%) and penicillin-streptomycin (1%) solution and maintained at 37 °C in a 5% CO_2_ incubator (Thermo Fisher Scientific, Waltham, MA, USA). Every two days, the whole medium was replaced. The cells were passaged every two or three days.

##### Cytotoxicity Assay

The cytotoxicity of N-acetyl-L-cysteine (NAC, as positive control) and JSP sub-fractions was evaluated using a CCK-8 assay kit [16]. RIN-m5F cells (100 µL/well) were inoculated in a 96-well plate and cultured for 24–48 h (37 °C) in the incubator. The solutions of NAC and JSP sub-fractions with concentrations of 100–1000 µg/mL were added. In the incubator, the culture plate was incubated for 24 h at 37 °C. Each hole was placed in 10 µL of CCK-8 solution and incubated for 1 h (37 °C). The absorbance was noted at 450 nm with an Epoch™ microplate spectrophotometer according to the CCK-8 manufacturer’s instruction.

##### Protective Effect on High-Glucose-Induced RIN-m5F Cell Injury

The protective capacities of JSP sub-fractions on RIN-m5F cells damaged by high glucose were determined using the procedure of Bei et al. (2019) with slight modification [17]. RIN-m5F cells were inoculated in a 96-well microplate (100 μL/well) and allowed to attach for 24–48 h (37 °C). Five groupings of cells were created. The normal group consisted of normal cells with a glucose concentration of 5 mM. The negative group contained cells damaged by 25 mM glucose. Cells that were treated with 100 µM NAC and 25 mM glucose generated a positive group. The JSP-1 group consisted of cells pretreated with low, middle, or high doses of JSP-1 (100, 500, and 1000 µM, respectively) followed by treatment with 25 mM glucose. The JSP-2 group included cells pre-incubated with different concentrations of JSP-2 (100, 500, and 1000 µM, respectively) and glucose (25 mM). All groups were cultured for 24 h (37 °C). The CCK-8 assay was used to determine cell proliferation (%).

### 2.5. Primary Structure Analysis of JSP Sub-Fractions

#### 2.5.1. Determination of Molecular Weight and Homogeneity

HPGPC with a Waters 1525 isocratic HPLC system coupled with a Waters 2414 refractive index detector (Waters Co., Milford, MA, USA) and a TSKgel 5000 column (PWXL 7.8 mm × 30 cm, TOSOH Corp., Tokyo, Japan) was used to compute the homogeneity and the MW of JSP sub-fractions. The mobile phase was 0.1 M NaNO_3_–0.01 M NaH_2_PO_4_ (pH 7.0) at a flow rate of 0.5 mL/min [18]. The 35 °C temperature was used for both the column and the detector. Dextran standards with varied average molecular weights (4.4, 9.9, 43.5, and 277 kDa) were applied to create a calibration curve, and the calibration curve of log (MW) vs. elution time (x) was as follows: log (MW) = −0.1292x + 8.6185 (r^2^ > 0.99).

#### 2.5.2. Analysis of Monosaccharide Composition

The monosaccharide content of each JSP sub-fraction from jasmine tea was determined by UPLC-MS/MS with PMP pre-column derivatization according to the slightly modified method [19]. In a sealed tube, 10 mg of sample was decomposed in 5 mL of 2 M trifluoroacetic acid (TFA) for 2 h (100 °C). After that, the hydrolyzed JSP-1 and JSP-2 were evaporated three times by using rotary vacuum evaporation and co-distillation with methanol to eliminate TFA. The hydrolyzed JSP-1, JSP-2, or standard solutions were mixed with 0.5 M PMP-methanol solvent for 2 h (70 °C), then neutralized with 0.3 M HCl. The solution was extracted with chloroform to filter the surplus chemicals. Prior to UPLC-MS analysis, the aqueous layer was filtered through a 0.45 μm membrane. Monosaccharide composition of JSP sub-fractions was performed on a Xevo TQ-S Micro triple quadrupole mass spectrometry (Waters, Milford, MA, USA) coupled to the Waters UPLC system (Waters) with an Agilent EC-C18 column (2.7 μm, 2.1 × 50 mm, Agilent Technologies Inc., Santa Clara, CA, USA). A total of 2 μL of filtrate prepared as above mentioned was injected into the HPLC apparatus. The mobile phase consisted of 20 mM phosphate buffer solution (pH 7.0) (A) and acetonitrile (B) at a flow rate of 0.4 mL/min. The gradient elution was conducted as follows: 0 min (86% A); 1 min (86% A); 7 min (81.5% A); 11 min (80% A); 13 min (40% A); 14.5 min (40% A); 14.6 min (86% A); 17 min (86% A). The column temperature was fixed at 35 °C. The operating parameters of mass spectrometry were as follows: scan modes, single ion monitor (SIM), spary voltage (2.0 kV), cone voltage (30 V), ion source temperature (150 °C), desolvation temperature (500 °C), desolvation gas flow (1000 L/h). The positive mode mass spectra of eluate were recorded at m/z 481.09, 495.10, 510.10, 511.08, and 525.06.

#### 2.5.3. FTIR and UV Analysis

Nicolet iS50 FTIR spectroscopy (Thermo Fisher Scientific, Waltham, MA, USA) was used to identify functional groups of the two JSP sub-fractions in the frequency range of 4000–400 cm^−1^ [20]. A total of 1 mg of dry sample and 100 mg of dry potassium bromide crystals were mixed and pressed into pellets, and the data were collected with the FTIR spectrophotometer. A UV analysis can identify homogeneous components of nucleic acid and proteins in polysaccharides. The sample solutions (1 mg/mL) were scanned on a Genesys 10S UV-Visible spectrophotometer (Thermo Fisher Scientific Inc., Waltham, MA, USA) from 190 to 400 nm.

#### 2.5.4. Congo Red Analysis

Congo red test was used to examine the conformational transition of JSP sub-fractions as the modified procedure [21]. In total, 1 mL of the sample (1 mg/mL) was diluted with an equal ratio of Congo red solution (80 μM). Then, various levels of 1 M NaOH solution were added to the final volume (0−0.5 M). The final volume of mixture was 4 mL. The maximum absorption wavelength (λmax) of the mixture was determined by Genesys 10S UV-Visible spectrophotometer (Thermo Fisher Scientific Inc., Waltham, MA, USA) in the range of 300–800 nm after standing for 5 min at room temperature. As a control, Congo red solution was mixed with the same concentration of NaOH.

#### 2.5.5. Analysis of NMR Spectroscopy

The sugar residues and their sequences in JSP-1 were acquired from NMR spectra. In a nutshell, 30 mg of JSP-1 was diluted in 0.5 mL of D_2_O. A Bruker AM-500 NMR spectrometer (Bruker BioSpin GmbH, Rheinstetten, Germany) was used to observe ^1^H NMR, ^13^C NMR, COSY, HSQC, and HMBC spectra of the JSP-1 fraction.

### 2.6. Statistical Analysis

All assays were performed in triplicate, with the exception of NMR spectroscopy analyses. The results are presented as means with standard deviations. The SPSS 25.0 software was used for one-way ANOVA (IBM, Chicago, IL, USA). Duncan’s test was used to detect the statistically significant differences (*p* < 0.05) between two groups.

## 3. Results and Discussion

### 3.1. Isolation and Purification of Crude JSP

**Figure 1:**
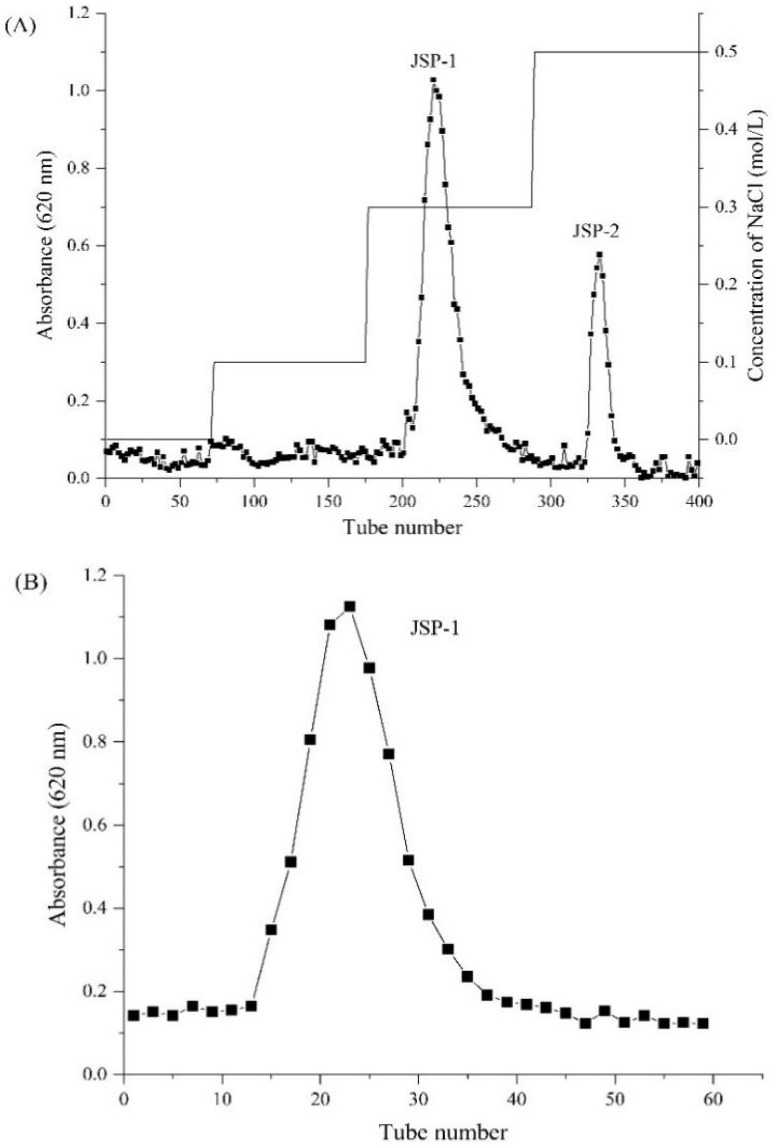

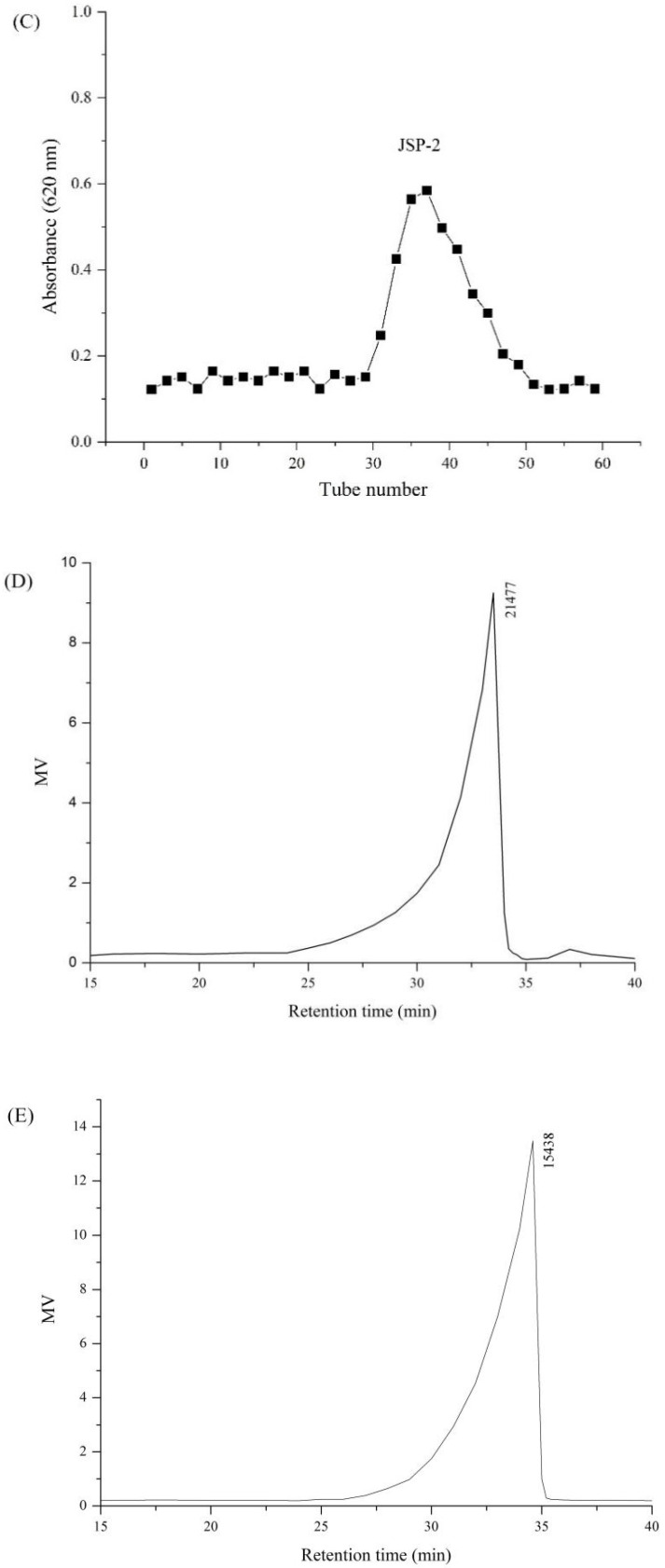
Elution profiles of *Jasminum sambac* flower polysaccharides (JSPs) on Diethylaminoethyl 52 (DEAE-52) cellulose and Sephadex G-100 column. (**A**) Crude JSP elution profile on DEAE-52 cellulose column; (**B**) elution profile of JSP-1 on Sephadex G-100 column; (**C**) elution profile of JSP-2 on Sephadex G-100 column; (**D**) high performance gel permeation chromatography (HPGPC) chromatogram of JSP-1; (**E**) HPGPC chromatogram of JSP-2.

The crude JSP was purified by DEAE-52 cellulose column eluted with ultra-pure water and different concentrations of NaCl, i.e., 0.1, 0.3, and 0.5 M. On the basis of their elution profiles (Figure 1A), two acidic sub-fractions (JSP-1 and JSP-2) were isolated and purified on the Sephadex G-100 column, showing a single peak in the spectra of JSP-1 and JSP-2 (Figure 1B,C), respectively. After dialysis and lyophilization, JSP-1 and JSP-2 accounted for 67.86% and 14.37% of crude JSP, respectively, as determined by the procedure of anthrone-sulfuric acid. Additionally, the HPGPC confirmed that the two sub-fractions showed symmetrical peaks, suggesting that each fraction was homogeneous (Figure 1D,E).

### 3.2. Functional Activities of JSP Sub-Fractions

#### 3.2.1. Antioxidant Activities

**Figure 2:**
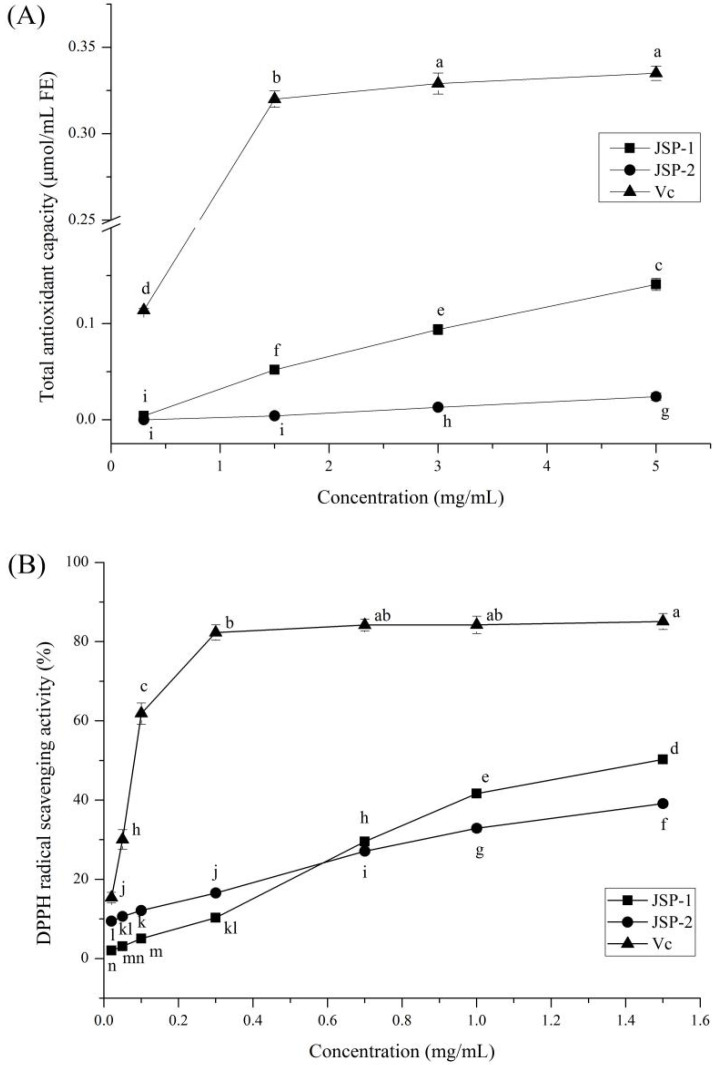

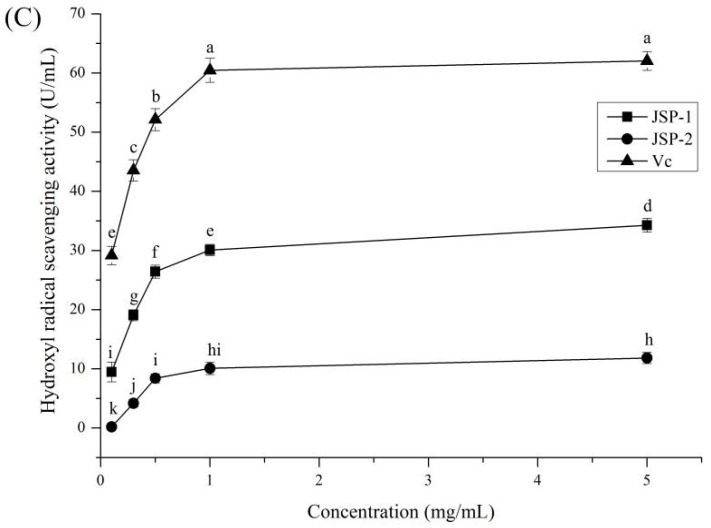
Antioxidant activities of *Jasminum sambac* flower polysaccharide (JSP) sub-fractions. (**A**) total antioxidant capacity (T-AOC), (**B**) 2,2-Diphenyl-1-picrylhydrazyl (DPPH), and (**C**) hydroxyl radical (•OH) scavenging activities. Values within each sample, denoted by various letters in the diagram, were statistically different, *p* < 0.05.

The existence of reactive free radicals in the human body over long periods of time can induce oxidative stress, which can lead to life-threatening diseases. Antioxidants are incredibly important because they may eliminate free radicals from the body, which can damage DNA and proteins. Antioxidant-rich foods should be consumed on a regular basis to avoid oxidative stress. As a result, the tests and the discussion focused on the antioxidant properties of JSP sub-fractions. Three methods of evaluation were used in this paper as: T-AOC and free radical scavenging (DPPH and •OH) assays. Concentration and activity profiles of JSP sub-fractions in these assays are presented in Figure 2, as compared to ascorbic acid.

The T-AOC assay is based on polysaccharides’ ability to convert ferric (Fe^3+^) to ferrous (Fe^2+^) ions. The T-AOC values of JSP sub-fractions increased in a concentration-dependent pattern (Figure 2A). JSP-1 and JSP-2 indicated different Fe^3+^-reducing power. JSP-1 exhibited a more potent reduction capacity of Fe^3+^ than that of JSP-2 at each concentration point. DPPH assay is normally conducted in an alcoholic solution as a result of its free radical with lipophilic properties. Hence, the relatively minimum content of JSP sub-fractions was applied in the current DPPH assay to reduce the development of precipitates or colloidal particles, resulting in an underestimation of polysaccharide activity or the conclusion of an inverse dose–response relationship [22]. The DPPH radical scavenging activity of JSP sub-fractions increased with concentrations over a range of 0.02–1.50 mg/mL, as shown in Figure 2B. The maximal DPPH radical scavenging activities of JSP-1, JSP-2, and ascorbic acid were 50.30%, 39.13%, and 85.11%, respectively, when the content neared 1.50 mg/mL. JSP-1 exhibited a more potent DPPH radical scavenging capacity than JSP-2 with concentration over 0.70 mg/mL. Among all types of free radicals, •OH is considered the most active and harmful free radical generated in the body. A positive association between concentration and •OH scavenging activity was confirmed in JSP-1 and JSP-2 (Figure 2C). The •OH scavenging activities of JSP-1, JSP-2, and ascorbic acid were as 34.30, 11.85, and 165.38 U/mL at 5.0 mg/mL, indicating that JSP-1 possessed stronger •OH scavenging capacity than JSP-2, which was lower than the positive control. According to the findings, JSP-1 has potential antioxidant properties and could be used as a source of bioactive compounds.

#### 3.2.2. Hypoglycemic Activities

Chronic hyperglycemia is mainly accountable for oxidative stress, which is the pivotal cause of increased oxidative stress damage [23]. Oxidative stress can directly cause islet cell damage, and the decreasing islet cell mass can contribute to a deteriorating diabetic state [24]. Recently, various plant polysaccharides effectively improved the antioxidant ability of diabetic rats and inhibited apoptosis of islet cells damaged by oxidative stress [25]. These polysaccharides, both edible and medicinal, have a wide range of applications in the food industry. Moreover, tea polysaccharides have the advantages of high safety and low toxicity in treating diabetes, including protecting islet cells, increasing the number of islet cells, promoting insulin secretion or release, and improving glucose metabolism. However, little attention was devoted to the protective effect of jasmine tea polysaccharides on islet cells. Thus, the high glucose-induced injury model was performed to evaluate the hypoglycemic effect in vitro of JSP sub-fractions, using NAC as the positive control.

**Figure 3:**
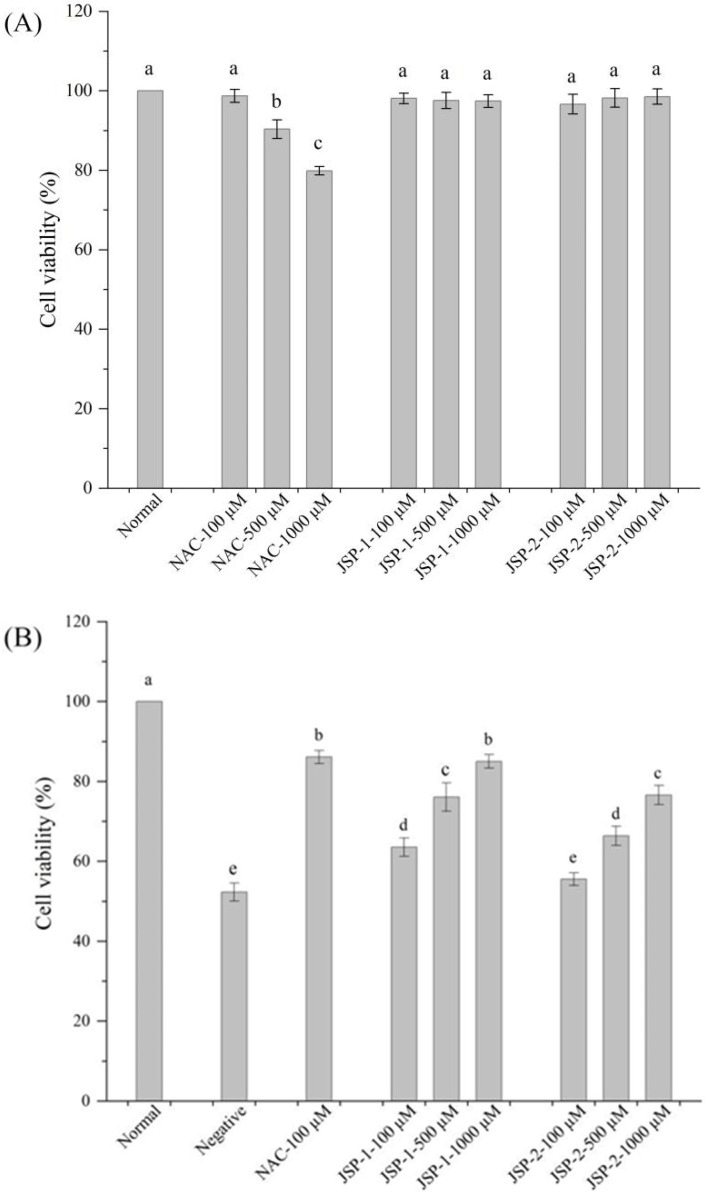
Hypoglycemic effect of JSP sub-fractions. (**A**) Cytotoxicity of JSP sub-fractions and N-acetyl-L-cysteine (NAC) on islet cells; (**B**) protective effect of JSP sub-fractions and NAC on high-glucose-induced hyperglycemic rat pancreatic islet (RIN-m5F) cells injury. Values within each sample, denoted by various letters in the diagram, were statistically different, *p* < 0.05.

From Figure 3A, the cytotoxic properties of JSP sub-fractions and NAC were assessed using RIN-m5F cells. JSP-1 and JSP-2 at the concentrations of 100−1000 μM exhibited no obvious toxicity on RIN-m5F cells. NAC showed slightly greater cytotoxicity of islet cells than JSP samples at 500 and 1000 μM (*p* < 0.05). JSP sub-fractions had significant protective effects on high-glucose damaged RIN-m5F cells (*p* < 0.05) at 100, 500, and 1000 μM (Figure 3B). Cell viability increased from 52.3 ± 2.3% (shown by negative group) to 85.0 ± 1.7 and 76.6 ± 2.4% for JSP-1 and JSP-2 at 1000 μM, respectively. Both JSP-1 and JSP-2 increased cell viability of RIN-m5F in a dose-dependent way. There were no significant differences (*p* > 0.05) in cell viability of RIN-m5f between JSP-1 at 1000 μM and NAC at 100 μM. JSP-1 with different concentrations exhibited significantly higher protective effects on high-glucose damaged RIN-m5F cells than JSP-2. Furthermore, JSP-1’s remarkable protective effect on islet cells could be attributed to its high antioxidant activity. 

### 3.3. Structural Analysis of JSP Sub-Fractions

#### 3.3.1. Monosaccharide Composition and Molecular Weight

According to Table 1, the MWs of JSP-1 and JSP-2 were found to be 18.4 kDa and 14.1 kDa, respectively, based on the calibration curve generated with standard dextrans. Furthermore, polysaccharides with MWs 4–100 kDa usually had low viscosity, which led to greater antioxidant and hypoglycemic activity since low viscosity means improved polysaccharide aqueous solution solubility [26]. The two JSP sub-fractions with low molecular weights fell within the above-mentioned range, thus showing antioxidant and hypoglycemic activities.

The two JSP sub-fractions were hetero-polysaccharides according to monosaccharide composition analyses based on hydrolysis and HPLC-MS profiles and consisted of galactose, rhamnose, xylose, arabinose, fucose, and mannose with molar ratios that differed considerably. JSP-1 was abundant in glucose (Table 1), although JSP-2 was not. Additionally, JSP-1 included 14.97% of glucuronic and galacturonic acid, but JSP-2 had 18.29% glucuronic and galacturonic acid, showing that JSP sub-fractions belong to acidic polysaccharides based on uronic acid level [27]. Moreover, variations in polysaccharide bioactivities were partially attributed to monosaccharide composition. The levels of galactose, rhamnose, arabinose, and galacturonic acid were maximum in JSP-1 compared to JSP-2. As per available findings that the antioxidant and the hypoglycemic activities of polysaccharides were linked with rhamnose, arabinose, and galacturonic acid, it makes a reasonable justification for the higher antioxidant and hypoglycemic activities of JSP-1 [28,29].

#### 3.3.2. FTIR and UV Analysis

**Figure 4:**
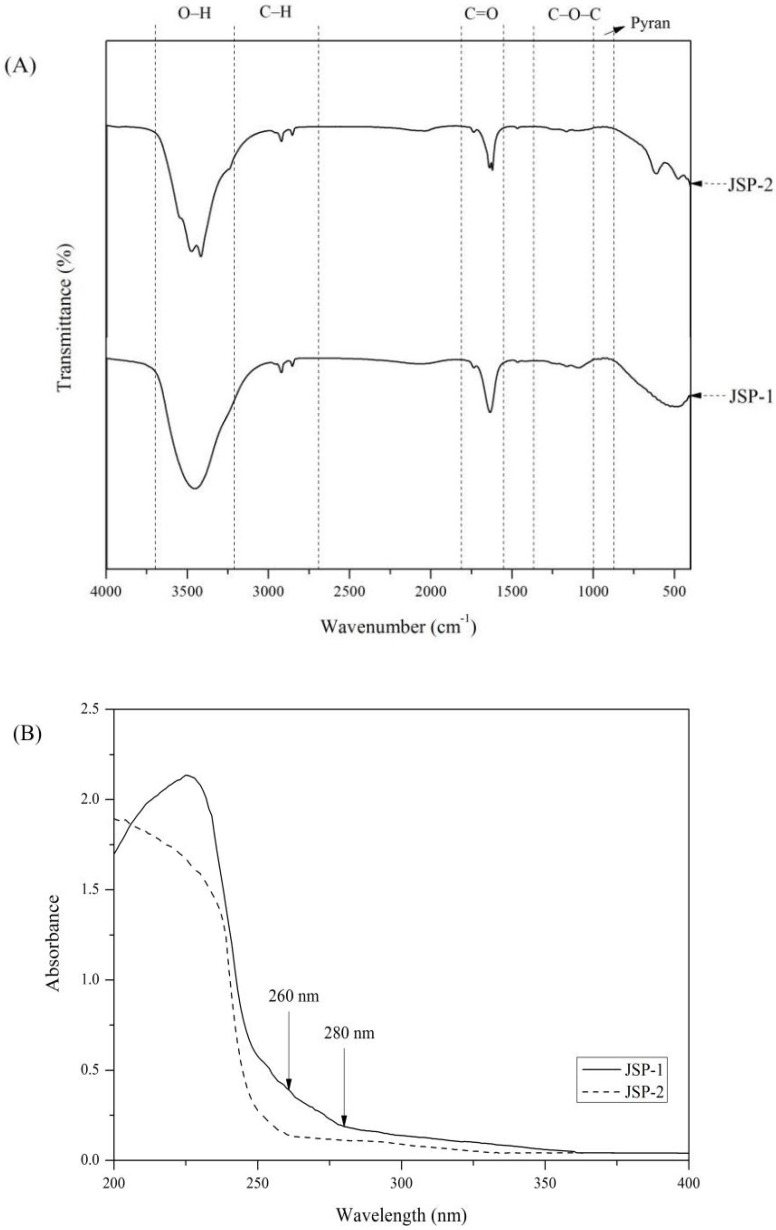
FTIR and UV analysis of JSP sub-fractions. (**A**) FTIR spectra of JSP-1 and JSP-2; (**B**) UV scan spectra of JSP-1 and JSP-2.

The FTIR spectrum of JSP-1 and JSP-2 is presented in Figure 4A. The JSP sub-fractions possessed typical absorption bands of polysaccharide. At 3400 cm^−1^, the O–H stretching vibration band was noticeably widened. In 2900–2800 cm^−1^, the stretching vibration peak of –CH_3_– and –CH_2_– was shown. There was no characterization absorption between 2500 and 1900 cm^−1^, indicating that no three or double bonds accumulated. Generally, the absorbances of the two JSP sub-fractions in the range of 1100–1000 cm^−1^ were assigned to C–O–C and C–OH, which indicated that sugar rings of JSP-1 and JSP-2 were pyranose rings [30]. Additionally, the stretching vibration of the carboxyl group (C=O) was linked to the absorbance range of 1700 cm^−1^ to 1600 cm^−1^, implying the presence of uronic acid [31]. The presence of uronic acid should be confirmed by NMR. Moreover, as shown in Figure 4B, there was no distinct absorption at the wavelength of 260 and 280 nm in the UV spectra from 200 nm to 400 nm, indicating that the two JSP sub-fractions were free of nucleic acid and protein.

#### 3.3.3. Congo Red Test

**Figure 5:**
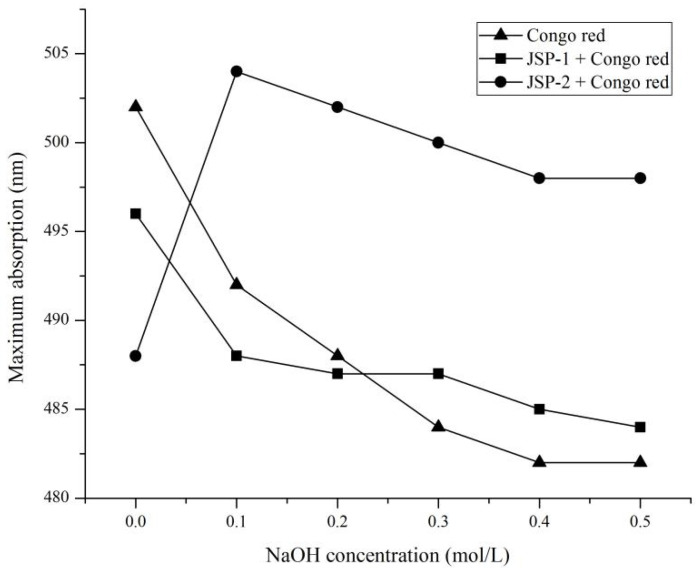
Analysis of triple helical conformation of JSP-1 and JSP-2.

In varying concentrations of alkaline solution, polysaccharides with a triple helical shape can form a complex with Congo red, generating a bathochromic shift of absorption maximum compared to pure Congo red in NaOH solution [32]. As the concentration of NaOH enhanced, the triple helical structure was destroyed, resulting in a drop in maximum absorption. The lack of a red shift and a significant decrease at higher NaOH concentrations (Figure 5) suggested that JSP-1 lacked a triple helical structure. When the concentration of NaOH was 0.1 M, JSP-2 showed a bathochromic shift, and the maximum absorption wavelength declined progressively as the concentration of NaOH increased, indicating that they had a triple helical structure in a slightly alkaline solution.

#### 3.3.4. NMR Studies

**Figure 6:**
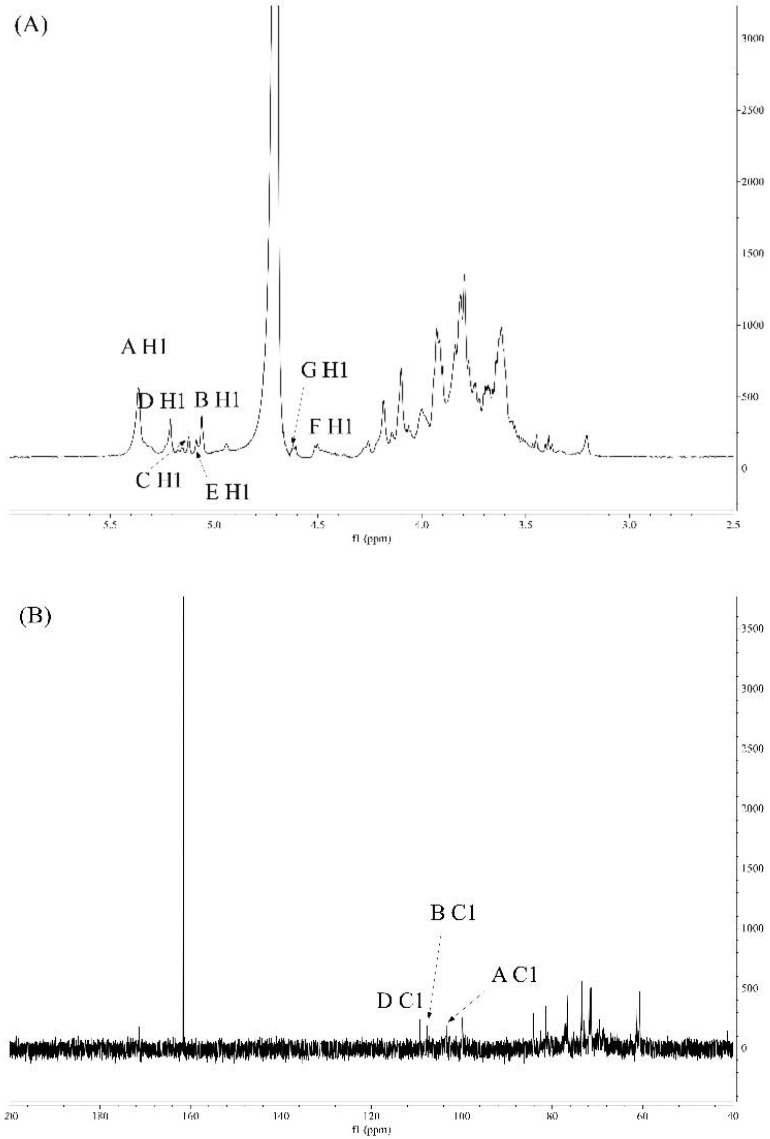
One-dimensional NMR studies of JSP-1. (**A**) ^1^H NMR spectrum; (**B**) ^13^C NMR spectrum.

**Figure 7:**
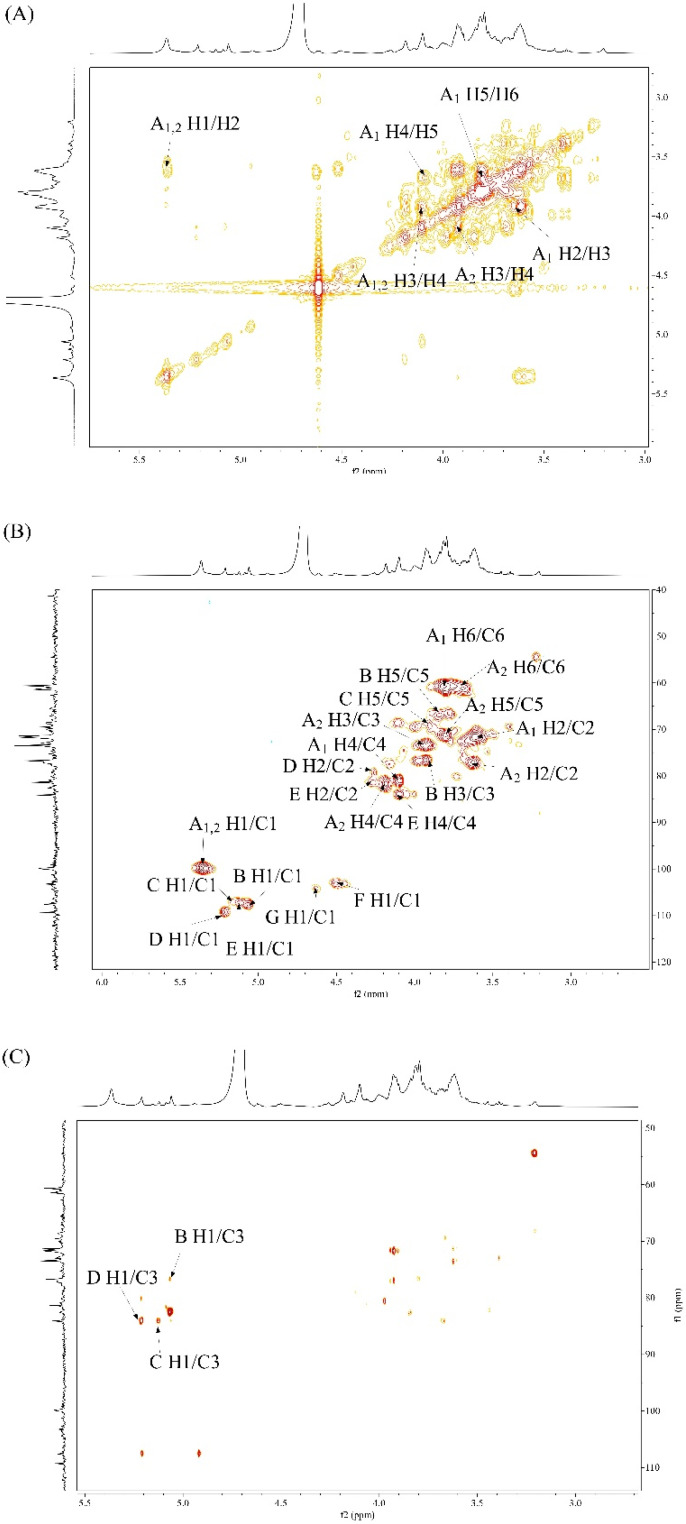
Two-dimensional NMR studies of JSP-1. (**A**) ^1^H-^1^H COSY spectrum; (**B**) HSQC spectrum; (**C**) HMBC spectrum.

By recording one-dimensional (^1^H and ^13^C) and two-dimensional (COSY, HSQC, and HMBC) NMR spectra, the sugar residues and their sequences of JSP-1 were identified. The primary chemical shifts of JSP-1 were discovered and are shown in Figure 6 and Figure 7 and Table 2 based on monosaccharide compositions, chemical signals, and previously published data.

The seven main anomeric protons were observed at 4.51, 4.62, 5.06, 5.08, 5.12, 5.21, and 5.36 ppm in ^1^H NMR spectrum, indicating the co-existence of α- and β-configurations in the JSP-1 fraction (Figure 6A). Anomeric protons in the ^13^C NMR spectrum were detected from 95 to 110 ppm (Figure 6B). The signals at 1.23 and 16.25 ppm were attributed to the presence of rhamnose, and the signal at 171.41 ppm confirmed the presence of uronic acid in JSP-1 [33], which corresponded to monosaccharide composition and FTIR analysis of JSP-1.

The COSY spectrum of JSP-1 (Figure 7A) demonstrated the coupling at 5.36/3.61, 3.61/3.93, 3.93/4.10, and 4.10/3.80 ppm. According to the HSQC spectrum of JSP-1 (Figure 7B), there were main five cross peaks at 5.36/99.75, 3.61/71.64, 3.93/76.75, 4.10/80.87, and 3.80/71.21 ppm. On the basis of literature, H1 of residue A1 was found to be 5.36 ppm, which was identified as 1,3-linked α-Gal*f* [34]. Meanwhile, cross peaks at 3.93/4.19 and 3.80/3.69 ppm were speculated in the COSY spectrum, and cross peaks at 3.63/77.07, 3.93/73.39, 4.19/81.79, and 3.69/61.43 ppm were demonstrated in the HSQC spectrum, which belonged to the characteristic anomeric signal of 1,2-linked α-Gal*f* [35]. For residues B, C, D, and E, chemical shifts at 5.06, 5.21, 5.12, and 5.08 ppm in ^1^H NMR spectrum were attributed to 1,5-linked α-Ara*f*, 1,3-linked α-Ara*f*, 1,3,5-linked α-Ara*f,* and T-linked α-Ara*f*, respectively [36,37,38]. Combined with the COSY spectrum, the H1–H5 signals of residues B, C, D, and E were unambiguously assigned (Table 2). The signal values for C1 to C5 were also assigned through HSQC spectra. Regarding the characteristic anomeric signals at 4.51 and 4.62 ppm in ^1^H NMR spectrum, residues F and G were probably xylopranoses in the β-anomeric configuration. Combining the COSY spectrum, the HSQC spectrum, and the published literatures, residues F and G corresponded to 1,2-linked β-Xyl*p* and 1,3-linked β-Xyl*p*. Moreover, the cross peak at 3.22/54.49 ppm in the HSQC spectrum confirmed the presence of the *O*-CH_3_ group in JSP-1.

Additionally, the HMBC spectrum (Figure 7C) was employed to confirm correlations between sugar residues. The cross signal at 5.06/67.60 ppm in the HMBC spectrum revealed that (1 → 5)-linked α-Ara*ƒ* was linked to *O*-5 of (1 → 5)-linked α-Ara*ƒ*. Cross signals at 5.06/76.75 and 5.36/77.07 ppm in the HMBC spectrum indicated that (1 → 5)-linked α-Ara*ƒ* could be linked to *O*-3 of (1 → 3)-linked α-Gal*ƒ*, while (1 → 3)-linked α-Gal*ƒ* was associated with *O*-2 of (1 → 2)-linked α-Gal*ƒ*. Additionally, cross signals at 5.06/84.12 and 5.21/84.21 ppm suggested that (1 → 5)-linked α-Ara*ƒ* might be linked to *O*-3 of (1 → 3)-linked α-Ara*ƒ*, while (1 → 3)-linked α-Ara*ƒ* was linked to *O*-3 of (1 → 3,5)-linked α-Ara*ƒ*.

Moreover, the type and the position of glycosidic linkage in polysaccharides-based products are directly linked to hypoglycemic action. The improved protective effects of active polysaccharides on islet cells are directly connected with the (1 → 3) glycosidic linkages [39]. Through NMR studies, JSP-1 contained (1 → 3)-linked α-Gal*ƒ*, (1 → 3)-linked α-Ara*ƒ* and (1 → 3)-linked β-Xyl*p*, which could be considered as the reason for antioxidant and hypoglycemic activities of JSP-1.

## 4. Conclusions

In the present study, two novel polysaccharide sub-fractions extracted from crude polysaccharides of jasmine tea were obtained by DEAE-52 cellulose and Sephadex G-100 chromatography, and their structural features were characterized. JSP-1 and JSP-2 had molecular weights of 18.4 kDa and 14.1 kDa, respectively. They are acidic hetero-polysaccharides with molar ratios of glucose, galactose, rhamnose, xylose, arabinose, and galacturonic acid that vary. The main structure of JSP-1 was composed of →1)-α-Gal*ƒ*-(3→, →1)-α-Gal*ƒ*-(2→, →1)-α-Ara*ƒ*-(5→, →1)-α-Ara*ƒ*-(3→, →1)-α-Ara*ƒ*-(3,5→, →1)-β-Xyl*p*-(2→ and →1)-β-Xyl*p*-(3→ residues. Moreover, JSP-1 and JSP-2 exhibited antioxidant activities and protective effects in RIN-m5F islet cells in a dose-dependent manner; however, JSP-1 exhibited better antioxidant and hypoglycemic activities than JSP-2. All data presented here suggested that the polysaccharides of jasmine tea could be a new source of natural antioxidants and hypoglycemics with potential value in healthy food. Antioxidant and anti-diabetic activities in vivo of novel bioactive polysaccharides from jasmine tea need to be investigated further.

## Figures and Tables

**Table 1:** JSP-1 and JSP-2 molecular weight and monosaccharide composition.

Sample	Molecular Weight (kDa)	Monosaccharide Composition (Molar Ratio)								
		Man	Glu	Gal	Rha	Fuc	Xyl	Ara	GlcA	GalA
JSP-1	18.4	0.06	1.14	4.69	1.00	0.18	9.92	13.79	0.10	4.09
JSP-2	14.1	0.29	-	3.80	1.00	0.17	8.27	11.85	0.14	5.05

Note: Man—mannose, Glu—glucose, Gal—galactose, Rha—rhamnose, Fuc—fucose, Xyl—xylose, Ara—arabinose, GlcA—glucuronic acid, GalA—galacturonic acid.

**Table 2:** ^1^H and ^13^C NMR chemical shifts (ppm) of JSP-1 recorded in D_2_O.

Sugar Residues			1	2	3	4	5	6
A_1_	1,3-linked α-Gal*f*	H	5.36	3.61	3.93	4.10	3.69	3.81
		C	99.75	71.64	76.75	80.87	72.85	60.66
A_2_	1,2-linked α-Gal*f*	H	5.36	3.63	3.93	4.19	3.80	3.69
		C	99.78	77.07	73.39	81.79	71.21	61.43
B	1,5-linked α-Ara*f*	H	5.06	4.10	3.99	4.19	3.85	-
		C	107.54	80.96	76.55	82.25	67.60	-
C	1,3,5-linked α-Ara*f*	H	5.12	-	4.26	4.06	3.90	-
		C	107.23	-	84.21	79.44	69.75	-
D	1,3-linked α-Ara*f*	H	5.21	4.19	4.10	4.24	-	-
		C	109.27	80.88	84.12	81.36	-	-
E	T-linked α-Ara*f*	H	5.08	4.27	4.19	4.00	3.69	-
		C	107.57	81.32	81.52	83.70	61.38	-
F	1,2-linked β-Xyl*p*	H	4.51	3.62	3.70	3.39	3.84	-
		C	103.26	71.44	75.23	69.46	60.66	-
G	1,3-linked β-Xyl*p*	H	4.62	3.63	3.75	4.07	3.84	-
		C	104.42	77.23	80.01	74.54	60.66	-

Note: “-” means signal was not detected.

## Data Availability

Not applicable.

## Abbreviations

Homo-nuclear chemical shift correlation spectroscopy (COSY), Fourier transform infrared spectroscopy (FTIR), high performance gel permeation chromatography (HPGPC), hetero-nuclear single quantum coherence (HSQC), hetero-nuclear multiple bond correlation (HMBC), *Jasminum sambac* flower polysaccharides (JSP), molecular weight (MW), nuclear magnetic resonance (NMR), total antioxidant capacity (T-AOC), ultra-performance liquid chromatography-tandem mass spectrometry (UPLC-MS), ultraviolet (UV).
